# Usefulness of complete blood count parameters to predict poor outcomes in cancer patients with febrile neutropenia presenting to the emergency department

**DOI:** 10.1080/07853890.2022.2031271

**Published:** 2022-02-17

**Authors:** Arom Choi, Incheol Park, Hye Sun Lee, Jinseok Chung, Min Joung Kim, Yoo Seok Park

**Affiliations:** a Department of Emergency Medicine, Yonsei University College of Medicine, Seoul, Republic of Korea; b Biostatistics Collaboration Unit, Yonsei Biomedical Research Institute, Yonsei University College of Medicine, Seoul, Republic of Korea

**Keywords:** Febrile neutropenia, mortality, serious medical complications, mean platelet volume, qSOFA, prediction model, nomogram

## Abstract

**Introduction:**

Febrile neutropenia (FN) is one of the major complications with high mortality rates in cancer patients undergoing chemotherapy. The Multinational Association for Supportive Care in Cancer (MASCC) risk-index score has limited applicability for routine use in the emergency department (ED). This study aimed to develop simplified new nomograms that can predict 28-day mortality and the development of serious medical complications in patients with FN by using a combination of complete blood count (CBC) parameters with quick Sequential Organ Failure Assessment (qSOFA).

**Methods:**

In this retrospective observational study, various models comprising qSOFA score and individual CBC parameters (red cell distribution width, delta neutrophil index, mean platelet volume (MPV)) were evaluated for association with outcomes by a multivariate logistic analysis. Subsequently, nomograms were developed for outcome prediction. The primary outcome was mortality at 28 days from ED presentation; the secondary outcome was the development of serious medical complications.

**Results:**

A total of 378 patients were included. Among the CBC parameters, only MPV was significantly associated with 28-day mortality and serious medical complications in patients with FN. The nomogram developed to predict 28-day mortality and serious medical complications showed good discrimination with area under the receiver-operating characteristic curve (AUC) values of 0.729 and 0.862 (95% CI, 0.780–0.943), respectively, which were not different from those of the MASCC score (0.814, 95% CI, 0.705–0.922; *p* = .07 and 0.921, 95% CI, 0.863–0.979; *p* = .11, respectively) in the validation set. The calibration of both nomograms demonstrated good agreement in the validation set.

**Conclusion:**

In this study, a novel prognostic nomogram using qSOFA score and MPV to identify cancer patients with FN with high risk of 28-day mortality and serious medical complications was verified and validated. Prompt management of fatal complications of FN can be possible through early prediction of poor outcomes with these new nomograms.

**KEY MESSAGES**
- Among the evaluated CBC parameters, only mean platelet volume was associated with 28-day mortality and serious medical complications in cancer patients with febrile neutropenia.
- A novel and rapid prognostic nomogram was developed using quick Sequential Organ Failure Assessment score and mean platelet volume to identify cancer patients with febrile neutropenia having high risk of 28-day mortality and serious medical complications.
- The nomogram developed to predict 28-day mortality and serious medical complications in patients with febrile neutropenia showed good discrimination and provides rapid patient evaluation that is especially applicable in the emergency department.

## Introduction

Febrile neutropenia (FN) is the major complications in cancer patients undergoing chemotherapy. Despite significant progress in the prevention and treatment of FN, the mortality rate in inpatients with FN ranges up to 20% [1–2]. Early prediction of poor outcomes and application of broad-spectrum antibiotics are crucial for cancer patients with immune-compromised conditions, especially in the emergency department (ED), to manage fatal complications and achieve maximum benefit from the prescribed chemotherapy [3–6].

Klastersky et al. developed the Multinational Association for Supportive Care in Cancer (MASCC) risk-index score in 2000 to recognise cancer patients with FN with a low risk of medical complications and who are therefore potentially suitable for outpatient clinic-based treatment [7]. An MASCC risk-index score ≥21, which was based on clinical characteristics, was recommended as the threshold for low risk. This index has been validated by various studies and used as a basis for several guidelines [8–10]. However, the MASCC risk-index score has limited utility for routine application in the ED, where patients with a high risk of complications and mortality should be identified [11]. Moreover, its subjectivity could be an issue because the ‘burden of illness’ was evaluated by attending physicians using visual analogue scales that measure symptom severity and physiologic reserve [7].

In 2016, the Sepsis-3 task force introduced the quick Sequential Organ Failure Assessment (qSOFA) score as a new method to identify among patients outside the intensive care unit (ICU) with suspected infection, those with a higher risk of poor outcomes. The qSOFA score can be easily obtained at the bedside to screen patients with infection who are likely to have a poor prognosis [12–13]. Kim et al. reported the predictive role of qSOFA for patients with FN who were admitted to the ICU. They showed that the qSOFA score was an independent predictive factor to identify sepsis and ICU admission in multivariate analysis [3]. However, the area under the receiver-operating characteristic curve (AUC) values of the qSOFA score for predicting sepsis, 28-day mortality, and ICU admission were 0.678, 0.651, and 0.715, respectively, which were inferior to those of the MASCC risk-index score (0.831, 0.856, and 0.835, respectively). Therefore, qSOFA alone may not be enough if it is not supported by other elements that increase the discriminative ability.

Furthermore, in the ED, promptly predicting deterioration of patients with FN using laboratory results such as the complete blood count (CBC) is difficult. Kim et al. introduced a scoring system to predict mortality in patients with sepsis and septic shock using red cell distribution width (RDW), delta neutrophil index (DNI), and platelet count [14]. According to the study, the AUC of this scoring system was 0.785, whereas those of lactate and SOFA score were 0.724 and 0.738, respectively. Furthermore, mean platelet volume (MPV) is known as a marker of platelet function and activation, related to inflammation and disease activity of chronic inflammatory disorders [15]. Another study showed that an increase in MPV during the first 72 h of admission was significantly associated with 28-day mortality in patients with sepsis and septic shock [16]. However, no studies have been performed using these laboratory tests in patients with FN.

Thus, the objective of this study was to develop simplified nomograms that can predict the prognosis of patients with FN who underwent chemotherapy by using a combination of CBC parameters along with qSOFA. The nomogram is a useful method since its statistical predictions can be represented visually; furthermore, this is a more exact tool than the conventional prediction model using odds ratios [17]. External validation of the nomogram was also performed.

## Materials and methods

### Study design and population

This study was a retrospective observational analysis of cancer patients aged over 18 years with FN who visited the ED in Severance Hospital, Yonsei University College of Medicine. FN was defined as a body temperature of over 38.0 °C with an absolute neutrophil count (ANC) of <500 cells/mm^3^ or a confirmed decrease in ANC from <1000 cells/mm^3^ to <500 cells/mm^3^ within 48 h [18]. Patients who signed a ‘Do not attempt resuscitation’ order and refused to be treated in an ICU were excluded. We also excluded patients without outcome data. This study was approved by the Institutional Review Board of Yonsei University College of Medicine, Severance Hospital (no. 4-2020-0095). The requirement for individual consent from patients for using their data was waived because of the retrospective study design.

### Data collection

The data of cancer patients admitted to the ED between May 2017 and August 2018 were retrieved retrospectively from the electronic medical record (EMR). The following data were obtained: past medical history, physical examination, vital signs at the ED visit, laboratory tests including CBC parameters, such as RDW, MPV, and DNI, possible infection sources, use of mechanical ventilation, use of vasopressors, application of continuous renal replacement therapy, ICU admission, and mortality. The MASCC and qSOFA scores were calculated using the available clinical information at the time of the ED visit. The primary outcome was mortality at 28 days from ED presentation. The secondary outcome was the development of serious medical complications, such as hypotension, respiratory failure, ICU admission, disseminated intravascular coagulation, confusion or altered mental state, congestive cardiac failure, bleeding severe enough to require transfusion, arrhythmia or ECG changes requiring treatment, or renal failure requiring investigation and/or treatment [7]. The final outcome of each febrile neutropenic episode was considered as the development of serious medical complications [18].

### Statistical analysis

All statistical analyses were performed using SAS version 9.4 (SAS Institute Inc., Cary, NC, USA) and R software version 3.4.4 for Windows (R foundation for statistical computing, Vienna, Austria). The results were presented as mean ± standard deviation (SD) or median (interquartile range) for continuous variables and frequencies (%) for categorical variables. For comparisons of continuous variables, we used independent two-sample t-tests if the data were normally distributed or the Mann–Whitney U test if the data were not normally distributed. The chi-squared test was used for categorical variables.

The study population was randomly allocated to either a training set or a validation set at a 7:3 ratio. In the training set, we created various types of combinations composed of qSOFA and individual CBC parameters such as MPV and then selected the models independently associated with primary or secondary outcomes by a multivariate logistic analysis method. Finally, the nomograms were developed for outcome prediction with the training set using variables included in the models. The performances of the nomograms were evaluated with respect to discrimination and calibration [19]. The predictive accuracy (discrimination) of the models was assessed by using the AUC values with their 95% confidence intervals (CIs), which quantifies the level of concordance between the predicted probabilities and the actual chance of the event of interest occurring. The DeLong method was used to compare the AUC for the predictive value of the new nomogram with those of the qSOFA and MASCC risk-index scores regarding the 28-day mortality and development of serious medical complications in patients with FN. Calibration of the nomograms determines how far the predicted probabilities are from the observed outcome frequencies using graphic representations (calibration curve). A plot along the 45-degree line would indicate a perfect calibration model in which the predicted probabilities are identical to the actual outcomes. The calibration curves are presented as calibration plots using the bootstrapping methods with 200 re-samples. Thereafter, the predictive accuracy and calibration of the new nomograms generated with the training set were tested in the validation set. All reported p-values were 2-sided, and results were considered statistically significant at *p* < .05.

## Results

**Figure 1.**
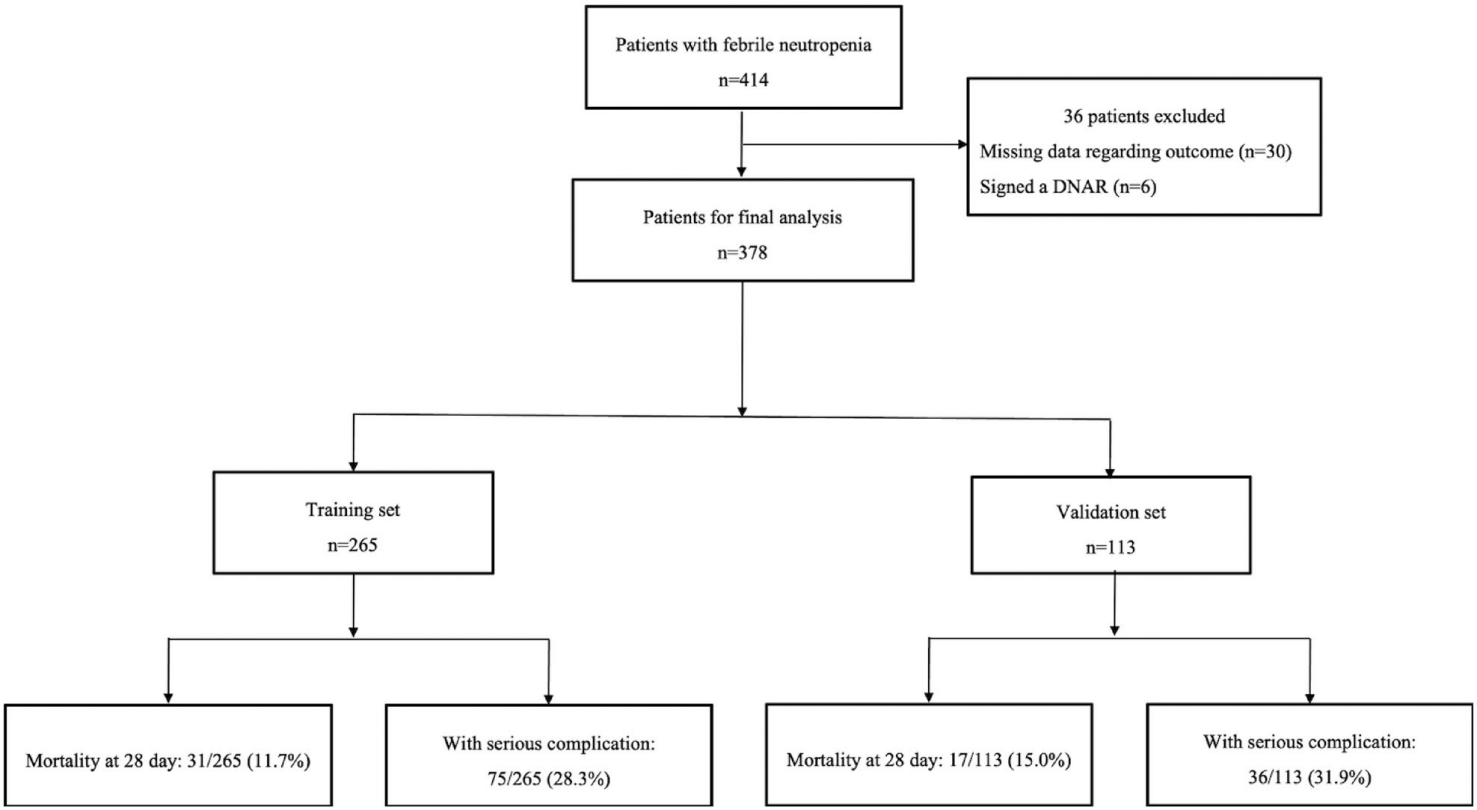
Patient flowchart. DNAR: ‘Do not attempt resuscitation’.

A total of 414 patient data, which showed FN after chemotherapy, were reviewed. Thirty patients whose outcome data were missing and six patients who signed a ‘Do not attempt resuscitation’ order were excluded. We included 378 patients in the final analysis, of whom 265 were randomly allocated to the training set and 113 to the validation set (Figure 1). All variables except ANC values were not significantly different between the training and validation set.

### 28-Day mortality

In the training set, 31 out of 265 (11.7%) patients died within 28 days after the ED visit. The baseline characteristics are summarised in Table 1. Among the three CBC parameters, the MPV values of those who died were higher than those of survivors (10.0 ± 1.7 vs. 8.8 ± 1.4; *p* < .001), while DNI and RDW values showed no differences between the groups. Further, the qSOFA and MASCC scores were significantly different between the groups (1.0 ± 0.9 vs. 0.3 ± 0.5; *p* < .001 and 14.3 ± 5.3 vs. 20.0 ± 4.4; *p* < .001, respectively). All CBC parameters were considered while generating models to predict 28-day mortality in patients with FN. Each CBC parameter was entered individually into the multivariate logistic regression analysis along with qSOFA among the patients in the training set (models 1–3). Table 2 summarises the results of the multivariate logistic regression analysis that revealed that only MPV was associated with 28-day mortality in patients with FN (*p* < .001).

**Table 1.** Patient demographics and clinical characteristics according to survival outcome at 28 days.

	Training set (*n* = 265)			Validation set (*n* = 113)		
	Survival (*n* = 234)	Death (*n* = 31)	*p* Value	Survival (*n* = 96)	Death (*n* = 17)	*p* Value
Age	58.1 ± 14.6	65.6 ± 12.4	.007	57.0 ± 15.4	63.5 ± 11.8	.10
Gender			.003			.02
Female	162 (69.2)	13 (41.9)		58 (60.4)	5 (29.4)	
Male	72 (30.8)	18 (58.1)		38 (39.6)	12 (70.6)	
SBP(mmHg)	115.1 ± 21.0	106.3 ± 25.6	.03	117.5 ± 24.6	107.2 ± 30.1	.13
PR(/min)	106.2 ± 18.8	111.9 ± 22.0	.12	109.2 ± 18.3	108.7 ± 18.8	.93
RR(/min)	17.2 ± 2.4	19.6 ± 4.5	.01	16.9 ± 3.0	17.9 ± 2.1	.20
BT(°C)	38.3 ± 0.8	38.4 ± 0.9	.76	38.2 ± 0.8	38.4 ± 0.6	.21
Laboratory test						
DNI(%)	5.8 ± 31.1	7.7 ± 13.3	.56	5.3 ± 8.6	2.3 ± 4.8	.05
MPV(fL)	8.8 ± 1.4	10.0 ± 1.7	<.001	8.7 ± 1.5	9.5 ± 2.4	.25
RDW(%)	14.9 ± 2.3	15.5 ± 2.2	.17	15.1 ± 2.5	15.7 ± 2.6	.40
WBC	1305.9 ± 4473.5	7598.4 ± 38085.5	.37	962.8 ± 558.4	3970.0 ± 12138.3	.32
ANC	172.2 ± 168.0	163.5 ± 173.3	.79	214.2 ± 225.8	298.7 ± 440.3	.45
Platelet(/1000)	128.4 ± 96.5	79.6 ± 93.8	.01	124.4 ± 93.0	80.2 ± 84.6	.07
CRP(mg/L)	96.6 ± 95.5	210.6 ± 113.8	<.001	106.8 ± 89.8	166.2 ± 117.9	.02
Procalcitonin(ng/mL)	3.9 ± 13.5	22.4 ± 34.8	.02	4.8 ± 12.6	9.6 ± 15.5	.26
Lactate(mmol/L)	2.0 ± 4.8	3.8 ± 3.4	.10	2.0 ± 2.4	3.1 ± 1.8	.19
Past history						
Cancer type			.02			.09
Solid	169 (72.2)	16 (51.6)		71 (74.0)	9 (52.9)	
Haematologic	65 (27.8)	15 (48.4)		25 (26.0)	8 (47.1)	
Pulmonary			.02			.70
No	205 (87.6)	22 (71.0)		84 (87.5)	14 (82.4)	
Yes	29 (12.4)	9 (29.0)		12 (12.5)	3 (17.6)	
Heart failure			.31			>.99
No	231 (99.1)	30 (96.8)		95 (99.0)	17 (100.0)	
Yes	2 (0.9)	1 (3.2)		1 (1.0)	0 (0.0)	
MASCC	20.0 ± 4.4	14.3 ± 5.3	<.001	19.8 ± 4.8	14.3 ± 4.8	<.001
qSOFA	0.3 ± 0.5	1.0 ± 0.9	<.001	0.3 ± 0.5	0.8 ± 0.8	.03

Variables are shown as mean ± standard deviation or number (percentage).

SBP: systolic blood pressure; PR: pulse rate; RR: respiratory rate; BT: body temperature; DNI: delta neutrophil index; MPV: mean platelet volume; RDW: red cell distribution width; WBC: white blood cell; ANC: absolute neutrophil count; CRP: C-reactive protein; MASCC: Multinational Association of Supportive Care in Cancer; qSOFA: quick Sequential Organ Failure Assessment.

**Table 2.** Multivariate analysis predicting 28-day mortality and serious medical complications in patients with febrile neutropenia from the training set.

	qSOFA + MPV		qSOFA + DNI		qSOFA + RDW	
	OR (95 % CI)	*p* Value	OR (95 % CI)	*p* Value	OR (95 % CI)	*p* Value
28-day mortality						
qSOFA	3.290 (1.922–5.632)	<.001	3.848 (2.202–6.724)	<.001	3.543 (2.096–5.990)	<.001
MPV	1.517 (1.194–1.929)	<.001				
DNI			1.001 (0.987–1.016)	.88		
RDW					1.073 (0.908–1.269)	.41
Serious medical complications						
qSOFA	16.187 (8.210–31.914)	<.001	18.236 (8.981–37.029)	<.001	17.158 (8.766–33.585)	<.001
MPV	1.339 (1.060–1.691)	.01				
DNI			0.998 (0.977–1.020)	.86		
RDW					1.167 (0.995–1.369)	.06

qSOFA: quick Sequential Organ Failure Assessment; MPV: mean platelet volume; DNI: delta neutrophil index; RDW: red cell distribution width; OR: odds ratio; CI: confidence interval.

**Figure 2.**
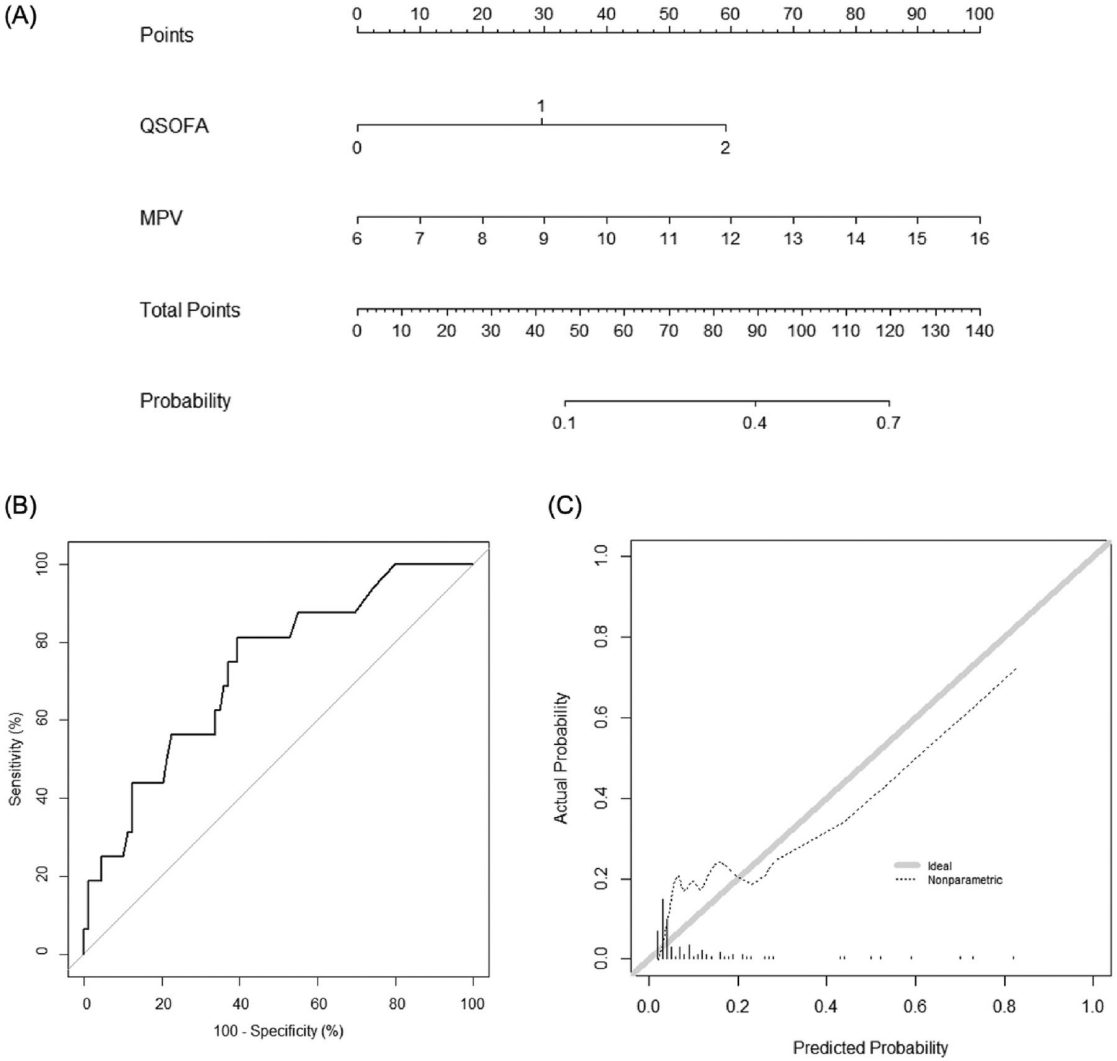
Nomogram for predicting 28-day mortality. (A) The novel nomogram for predicting 28-day mortality, developed using the training data set, is shown. (B) The discriminative ability of the nomogram is good, with an area under the receiver-operating characteristic curve of 0.729 (95% confidence interval: 0.598–0.861). (C) The calibration plot of the prediction model indicates good agreement between the predicted and observed probabilities of survival discharge. qSOFA: quick Sequential Organ Failure Assessment; MPV: mean platelet volume.

A new nomogram incorporating MPV and qSOFA score was established (Figure 2(A)); it illustrated that MPV was the largest contributor to prognosis in patients with FN. By calculating the total number of points and locating it on the total point scale, we were easily able to draw a straight line down to estimate the predicted probability of 28-day mortality. The AUC of the new nomogram was 0.834 (95% CI, 0.762–0.905), which was higher than that of the qSOFA score (0.718, 95% CI, 0.613–0.824; *p* = .002) and similar to that of the MASCC score (0.808, 95% CI, 0.718–0.898; *p* = .55). In the validation set, discrimination was good with an AUC value of 0.729 (95% CI, 0.598–0.861), which was not different to those of the qSOFA or MASCC scores (0.710, 95% CI, 0.580–0.841; *p* = .63 and 0.814, 95% CI, 0.705–0.922; *p* = .07, respectively) (Figure 2(B)). The calibration plot of the nomogram demonstrated a good agreement between the predicted and observed probabilities of survival discharge in the validation set (Figure 2(C)).

### Serious medical complications

**Figure 3.**
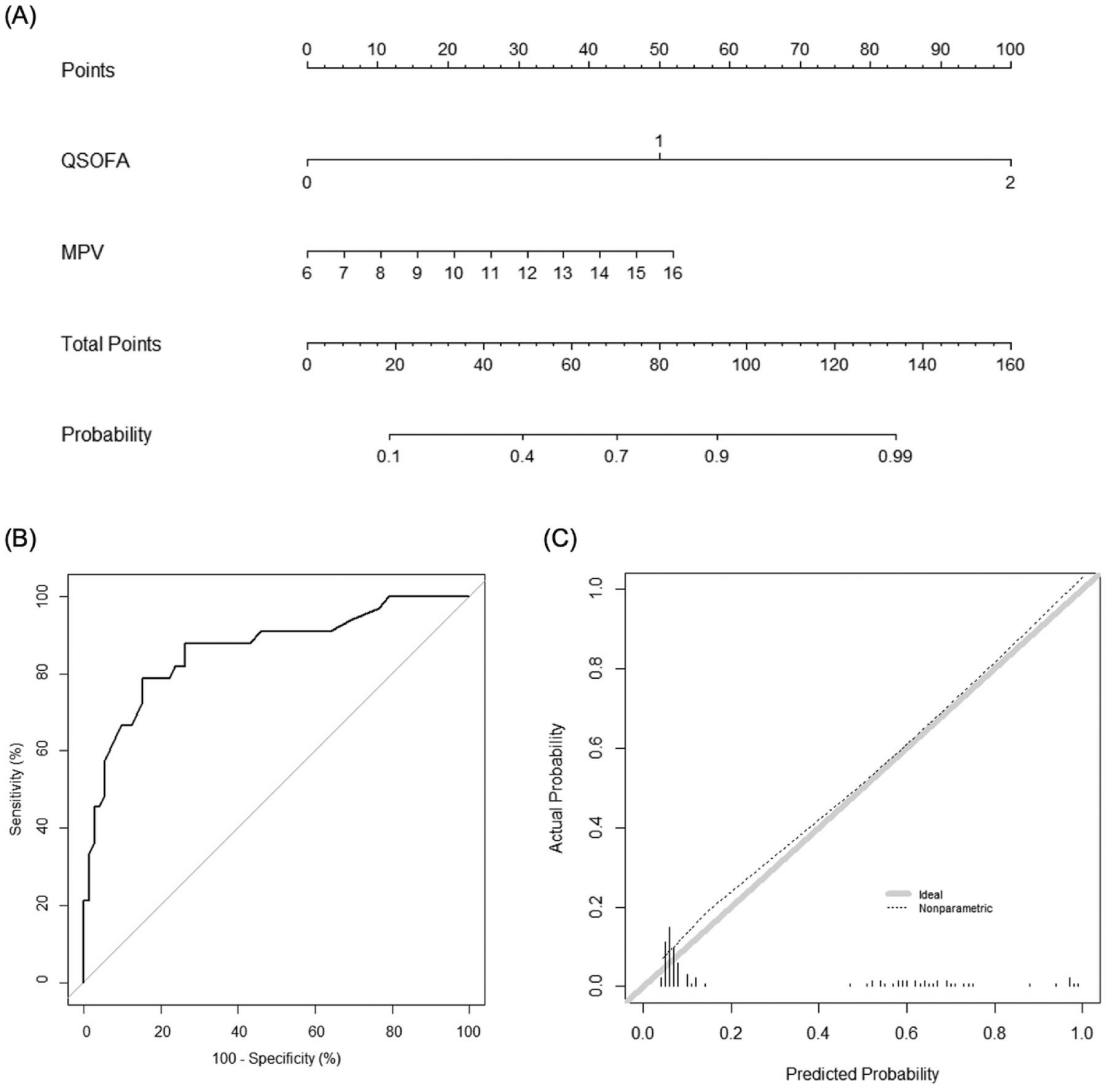
Nomogram for predicting the development of serious medical complications. (A) The novel nomogram for predicting the development of serious medical complications, developed using the training data set, is shown. (B) The discriminative ability of the nomogram is good, with an area under the receiver-operating characteristic curve of 0.862 (95% confidence interval: 0.780–0.943). (C) The calibration plot of the prediction model indicates good agreement between the predicted and observed probabilities of the development of serious medical complications. qSOFA: quick Sequential Organ Failure Assessment; MPV: mean platelet volume.

In the training set, 75 out of 265 patients (28.3%) experienced serious medical complications. The baseline characteristics and variables found to be associated with the development of serious medical complications are summarised in Table 3. Among the three CBC parameters, MPV and RDW, but not DNI, showed statistically significant differences between patients with and without serious medical complications (*p* < .001 and *p* = .01, respectively). The qSOFA and MASCC scores were also significantly different between the groups (1.0 ± 0.7 vs. 0.1 ± 0.3; *p* < .001, and 14.3 ± 4.5 vs. 21.3 ± 3.4; *p* < .001, respectively). Using multivariate logistic regression based on the combination of individual CBC parameters and the qSOFA scores, we found that MPV was associated with the occurrence of serious medical complications (*p* = .01), while RDW was not (*p* = .06) (Table 2). Similarly, we developed a new nomogram for predicting the development of serious medical complications (Figure 3(A)), wherein MPV was found to be the most contributing factor (Figure 3(A)). The AUC value of the nomogram was 0.894 (95% CI, 0.846–0.942). Further, in the validation set, discrimination was good with an AUC value of 0.862 (95% CI, 0.780–0.943), which was higher than that of the qSOFA score (0.814, 95% CI, 0.729–0.899; *p* = .03) and similar to that of the MASCC score (0.921, 95% CI, 0.863–0.979; *p* = .11) (Figure 3(B)). The calibration plot of the nomogram demonstrated good agreement between the predicted and observed probabilities of the development of serious medical complications in the validation set (Figure 3(C)).

**Table 3.** Patient demographics and clinical characteristics associated with the development of serious medical complications.

	Training set (*n* = 265)			Validation set (*n* = 113)		
	No (*n* = 190)	Yes (*n* = 75)	*p* Value	No (*n* = 77)	Yes (*n* = 36)	*p* Value
Age	56.8 ± 14.5	64.5 ± 13.4	<.001	54.6 ± 15.1	65.1 ± 12.5	<.001
Gender			<.001			<.001
Female	137 (72.1)	38 (50.7)		53 (68.8)	10 (27.8)	
Male	53 (27.9)	37 (49.3)		24 (31.2)	26 (72.2)	
SBP(mmHg)	117.8 ± 17.9	104.8 ± 27.3	<.001	120.7 ± 24.1	105.7 ± 26.2	.003
PR(/min)	105.0 ± 17.3	111.8 ± 23.0	.02	108.9 ± 17.9	109.6 ± 19.4	.85
RR(/min)	17.0 ± 1.9	18.9 ± 4.1	<.001	16.6 ± 2.6	18.1 ± 3.2	.007
BT(°C)	38.2 ± 0.7	38.5 ± 1.0	.05	38.1 ± 0.8	38.3 ± 0.9	.27
Laboratory test						
DNI(%)	5.9 ± 34.1	6.2 ± 10.8	.90	4.8 ± 7.4	5.1 ± 9.8	.84
MPV(fL)	8.7 ± 1.3	9.4 ± 1.6	<.001	8.5 ± 1.0	9.7 ± 2.4	.007
RDW(%)	14.7 ± 2.3	15.5 ± 2.4	.01	14.8 ± 2.5	16.0 ± 2.4	.02
WBC	1427.7 ± 4951.1	3598.3 ± 24488.2	.45	1012.7 ± 562.9	2276.1 ± 8374.1	.37
ANC	169.5 ± 154.0	175.3 ± 201.1	.82	212.2 ± 208.3	258.3 ± 366.5	.49
Platelet(/1000)	136.8 ± 100.3	87.0 ± 79.3	<.001	135.7 ± 97.3	79.4 ± 69.1	<.001
CRP(mg/L)	90.3 ± 88.8	159.6 ± 123.1	<.001	97.4 ± 85.3	155.0 ± 107.5	.003
Procalcitonin(ng/mL)	1.5 ± 6.3	14.2 ± 28.0	<.001	0.7 ± 1.2	15.1 ± 19.3	.002
Lactate(mmol/L)	2.1 ± 5.8	2.7 ± 2.6	.44	1.3 ± 0.8	3.5 ± 3.0	.002
Past history						
Cancer type			.03			.04
Solid	140 (73.7)	45 (60.0)		59 (76.6)	21 (58.3)	
Haematololgic	50 (26.3)	30 (40.0)		18 (23.4)	15 (41.7)	
Pulmonary			.10			>.99
No	167 (87.9)	60 (80.0)		67 (87.0)	31 (86.1)	
Yes	23 (12.1)	15 (20.0)		10 (13.0)	5 (13.9)	
Heart failure			.19			>.99
No	189 (99.5)	72 (97.3)		76 (98.7)	36 (100.0)	
Yes	1 (0.5)	2 (2.7)		1 (1.3)	0 (0.0)	
MASCC	21.3 ± 3.4	14.3 ± 4.5	<.001	21.5 ± 3.4	13.5 ± 4.2	<.001
qSOFA	0.1 ± 0.3	1.0 ± 0.7	<.001	0.2 ± 0.4	0.9 ± 0.7	<.001

Variables are shown as mean ± standard deviation or number (percentage).

SBP: systolic blood pressure; PR: pulse rate; RR: respiratory rate; BT: body temperature; DNI: delta neutrophil index; MPV: mean platelet volume; RDW: red cell distribution width; WBC: white blood cell; ANC: absolute neutrophil count; CRP: C-reactive protein; MASCC: Multinational Association of Supportive Care in Cancer; qSOFA: quick Sequential Organ Failure Assessment.

## Discussion

This study investigated the predictive performance of a new prognostic model developed using a combination of CBC parameters and the qSOFA score in FN to predict 28-day mortality and serious medical complications in cancer patients presenting at the ED. Although the MASCC risk-index score has been validated under various conditions [20–22], it has limited utility especially in the ED due to its subjective approach in evaluating the ‘burden of illness’ and its nature of identifying mainly low-risk patients suitable for outpatient management [23]. Thus, a rapid and objective risk stratification tool should be developed and applied for use in the ED. This study revealed that when MPV was added to the qSOFA score, contrary to other CBC parameters such as DNI and RDW, the predictive performance was better than that of the qSOFA score and similar to that of the MASCC score. The qSOFA scoring system, developed by the Sepsis-3 task force, is a simple bedside measure used to identify patients outside the ICU with suspected infection who may have poor outcomes. It contains three different variables: altered mental status, respiratory rate (RR) ≥22/min, and SBP ≤100 mmHg. Patients showing at least two of these criteria were considered more likely to have sepsis [24]. It showed a discriminative ability superior to that of the systemic inflammatory response syndrome (SIRS) criteria for predicting mortality in patients with sepsis [13]. Kim et al. revealed that the qSOFA score showed superior discriminative ability to SIRS criteria, but inferior to the MASCC score in predicting sepsis, 28-day mortality, and ICU admission in patients with FN [3]. Therefore, an additional variable is needed to improve the performance of qSOFA in predicting poor outcomes of FN.

Furthermore, although several studies highlighted the utility of laboratory results as a predictive factor [25–26], no studies have used CBC parameters to predict the prognosis of patients with FN. A new prognostic model using CBC parameters, which are rapidly and cost-effectively measured, is reasonable for use in the EDs in general. MPV are not only an indicator of haemostasis and thrombosis but are also thought to play a critical role in inflammatory responses [27–29]. Platelets are known to change their structure and function from inactive to active platelets owing to physiologic signals [30]. As MPV reflects platelet size and activity, which are associated with inflammatory and thrombotic processes, it can be a marker of platelet activation [31]. Several studies suggest that elevated MPV is associated with an increased risk of mortality in critically ill patients [32–33]. Our study, conducted in the EDs, showed that a new prognostic model that included MPV with qSOFA revealed a poor prognosis of patients with FN.

Cancer patients receive platelet concentrate transfusion to manage disease course for several reasons since low platelet counts frequently lead to bleeding complications. A platelet concentrate transfusion might affect CBC parameter and platelet indices. In this study, 77/265 patients in the training set and 28/113 patients in the validation set received platelet concentrate transfusion within 1 week of FN encounter. MPV were 9.751 ± 1.717 and 8.590 ± 1.194 in patients who received platelet concentrates transfusion and who did not, respectively, in the training set (*p* = <.001). In the validation set, MPV were 9.748 ± 2.508 and 8.567 ± 1.142 in patients who received platelet concentrate transfusion and who did not, respectively (*p* = .025). To identify the difference in the predictive performance of the nomogram to predict 28-days mortality depending on the administration of platelet concentrate transfusion, we performed AUC subgroup comparison. The AUC of the nomogram for the subgroup with platelet concentrate transfusion was 0.795 (98% CI 0.664–0.927), while for the subgroup without transfusion was 0.844 (95% CI 0.753–0.934). There was no statistically significant difference between the two subgroups (*p* = .55), and no significant difference was observed between the entire cohorts and each subgroup with and without transfusion (*p* = .57, 0.83, respectively). Likewise, for AUC comparing the predictive performance of the nomogram of platelet concentrate transfusion administration on serious medical complications, the nomogram for the platelet concentrate transfusion subgroup was 0.837 (95% CI 0.734–0.941), while the subgroup without transfusion was 0.913 (95% 0.856–0.970). There was no statistically significant difference in the two subgroups (*p* = .21), and no significant difference was shown between the entire cohorts and each subgroup with and without transfusion (*p* = .28, 0.51, respectively). Therefore, the performance of nomogram to predict adverse outcome of FN was not associated with platelet concentrates transfusion, though MPV might be affected by platelet concentrates transfusion.

Elevated MPV was related to decreased renal function in a previous study, although the mechanism of MPV change in patients with decreased renal function is not fully understood. Ucar et al. suggested that the shortened lifespan of platelets in uraemic conditions could have stimulated platelet production [34]. Thus, the correlation between the estimated glomerular filtration rate (eGFR) and MPV was analysed, and the results showed a negligible correlation between eGFR and MPV (for eGFR (MDRD); R = −0.224, for eGFR (CKD-EPI); R = −0.257, all *p* < .001) [35]. Therefore, the difference in patients’ eGFR in this study was not considered to affect MPV value significantly.

Moreover, cancer types can be important in predicting prognosis of FN when MPV is used as a predictive factor. Previous studies identified the role of MPV in predicting disease course in various types of malignancy. MPV has prognostic value in many types of solid tumours and haematologic malignancies [36–39]. However, it is difficult to identify the different MPV impacts and mechanisms of action on the prognosis for each cancer type. In this study, for patients with solid tumours, the AUC of the nomogram to predict 28-days mortality was 0.862 (95% CI 0.787–0.936), which was not statistically different from the nomogram for the entire cohort [0.834 (95% CI 0.762–0.905), *p* = .46]. Likewise, for patients with haematologic malignancies, the AUC of the monogram was 0.759 (95% CI 0.614–0.905) and was not statistically different from the entire cohort’s nomogram (*p* = .32). For predicting serious medical complications development, the AUC of the monogram was 0.927 (95% CI 0.876–0.979) in the subgroup with solid tumours, which was not significantly different from the nomogram derived from the entire cohort [0.894 (0.846–0.942), *p* = .20]. Moreover, for patients with haematologic malignancies, the AUC of the monogram was 0.817(0.703–0.930), which was not different from the nomogram for entire cohort (*p* = .18). Although the nomogram developed in this study seemed to show a slightly better performance in predicting adverse outcome of FN in patients with solid tumours, the prognostic performance of the nomogram for the solid tumour’s subgroups and haematologic malignancies was not statistically different (28-days mortality; *p* = .09, serious medical complication; *p* = .16). In most solid tumours, the association between platelet sizes and prognosis is explained by activated platelets releasing chemokines, proteolytic factors, and growth factors for growth and tumour invasion. In addition, platelets are involved in tumour cells aggregation, which protects them from the immunologic system [40, 41]. Conversely, in haematological malignancy, MPV is more likely to associate with the extent of inflammation [42]. Cornillie et al. suggested that the release of platelets with small sizes from the bone marrow with haematologic malignancy increases the secretion of pro-inflammatory cytokines, which hinders megakaryopoiesis activity and accelerates activated platelets consumption [43]. Thus, one explanation for the different model performances is that several platelet mechanisms play a slightly different role in each cancer type.

The major advantage of the prognostic nomogram developed in our study compared to the MASCC or other models from previous study is the easier accessibility owing to both simple laboratory results and bedside patient evaluation. Previous studies revealed that several biomarkers could be a prognostic factor to predict outcomes of FN. According to Combariza et al., C-reactive protein (CRP) added to the MASCC model was useful in predicting mortality of FN in haematological malignancies [44]. Moreover, Ahn et al. introduced a new prognostic model including procalcitonin as prognostic factor of FN, which classifies patients for poor outcomes and bacteraemia. The model showed higher specificity and negative predictive values than the MASCC model [23]. In this study, the CRP of those who died within 28 days was higher than survivors (210.6 ± 113.8 vs. 96.6 ± 95.5; *p* < .001), and procalcitonin of those who died was also higher than survivors (22.4 ± 34.8 vs. 3.9 ± 13.5; *p* = .02). Moreover, CRP and procalcitonin also varied significantly between patients with and without serious medical complications (159.6 ± 123.1 vs. 90.3 ± 88.8; *p* < .001, and 14.2 ± 28.0 vs. 1.5 ± 6.3; *p* < .001, respectively). In this study, we developed a simple model for rapid prediction of poor outcome of FN at ED presentation. CBC is rapid, easily accessible, cost-effective, and repeatable. CBC parameters such as MPV as a prognostic factor would allow a quick assessment of the patient at the time of diagnosis at the ED since CBC parameters can be obtained without any additional laboratory test in most hospitals. Besides, qSOFA was used as the baseline tool for evaluating FN patients since qSOFA is one of the easiest methods at the bedside. The nomogram provides rapid patient evaluation that is especially applicable in the ED, and its discriminative ability was shown to be accurate enough to be approximate to the MASCC risk-index score statistically.

This study has some limitations. First, as this study is retrospective in nature, selection bias may have distorted the results. Second, generalisability of the study may be restricted due to the enrolment of patients in a single centre. Third, we did not analyse the effect of different regimens and the intensity of chemotherapy on the outcome, and these conditions could be possible confounders. Thus, further research is needed to identify the association between these factors and the prognostic performance of the nomogram.

In this study, we have verified and validated a novel and rapid prognostic nomogram to identify cancer patients with FN who have a high risk of 28-day mortality and serious medical complications. Early and accurate prediction enables prompt management of fatal complications of chemotherapy through close monitoring and rapid application of proper treatments such as antibiotics and source management. Furthermore, the accurate disposition of patients can be possible, particularly in ED. External validation and prospective multicentre studies are required to confirm the performance of our nomogram for future research.

## Data Availability

The data acquisition of the present study was approved by the Ethics Committee of Severance Hospital. Datasets used or analysed during the current study are available from the corresponding author on reasonable request.

## Abbreviations

**Abbreviations**
ANC: absolute neutrophil count
AUC: receiver-operating characteristic curve
BT: body temperature
CBC: complete blood count
CI: confidence interval
CRP: C-reactive protein
DNAR: Do not attempt resuscitation
DNI: delta neutrophil index
ED: emergency department
eGFR: estimated glomerular filtration rate
EMR: electronic medical record
FN: febrile neutropenia
ICU: intensive care unit
MASCC: the Multinational Association for Supportive Care in Cancer
MPV: mean platelet volume
OR: odds ratio
PR: pulse rate
qSOFA: quick Sequential Organ Failure Assessment
RDW: red cell distribution width
RR: respiratory rate
SBP: systolic blood pressure
SD: standard deviation
WBC: white blood cell
